# Trouble sleeping inside: a cross-sectional study of the prevalence and associated risk factors of insomnia in adult prison populations in England

**DOI:** 10.1016/j.sleep.2016.12.018

**Published:** 2017-04

**Authors:** Lindsay H. Dewa, Lamiece Hassan, Jenny J. Shaw, Jane Senior

**Affiliations:** a NIHR Imperial Patient Safety Translational Research Centre, Imperial College London, London, UK; b Division of Imaging, Informatics and Data Sciences, The University of Manchester, Manchester, UK; c Division of Psychology and Mental Health, The University of Manchester, UK

**Keywords:** Insomnia, Sleep, Prevalence, Risk factors, Prisons

## Abstract

**Objective:**

To investigate the prevalence of insomnia and identify associated demographic, clinical and forensic risk factors in adult prisoners in England.

**Methods:**

A cross-sectional study of 237 prisoners aged 18–72 years, across two male prisons and one female prison in North England. We used the Sleep Condition Indicator to measure probable DSM-V insomnia disorder (ID) and the Pittsburgh Sleep Quality Index to examine sleep quality. Multiple demographic, sleep, clinical and forensic self-reported measures were recorded to identify any associations with insomnia.

**Results:**

Overall, the prevalence of possible DSM-V ID was 61.6% (95% CI, 55.5%–67.8%). Subjective poor sleep quality was reported by 88.2% (95% CI, 84.1%–92.3%). Seven in ten (70.6%) female prisoners had possible DSM-V ID (95% CI, 64.8%–76.4%). Multivariable logistic regression analysis, adjusting for gender and age, indicated odds of having possible ID in prison were increased for the following factors: history of physical ill-health (OR = 3.62, 95% CI, 1.31–9.98); suicidality (OR = 2.79, 95% CI, 1.01.7.66), previously asked for help for insomnia (OR = 2.58, 95% CI, 1.21–5.47), depression (OR = 2.06, 95% CI 1.31–3.24), greater endorsement of dysfunctional beliefs about sleep (OR = 1.50, 95% CI, 1.21–1.87), poor sleep hygiene (OR = 1.11, 95% CI, 1.04–1.19), and problematic prison environment (eg, noise, light or temperature) (OR = 1.07, 95% CI, 1.02–1.12).

**Conclusions:**

For the first time we have established the prevalence and associated factors of insomnia in a large sample of adult English prisoners. ID and poor sleep quality are common, especially in female prisoners. These findings emphasize/amplify the need for dedicated treatment pathways to improve screening, assessment and treatment of insomnia in prison.

## Highlights

• Possible insomnia disorder was highly prevalent in prisoners and more likely in women prisoners.
• Insomnia was associated with a problematic prison environment, depression, suicidality and physical-ill health.
• Insomnia was associated with poor sleep hygiene and maladaptive beliefs about sleep.
• No association was found between insomnia and substance misuse.
• The high prevalence endorses the importance of future research on treatment targeting insomnia in prisoners.

## 1 Introduction

Insomnia is defined as having difficulty initiating or maintaining sleep, or experiencing early morning awakenings, with resultant daytime impairment [1], [2], [3]. It is the most common sleep disorder in the general population [4]. Prevalence estimates vary, ranging between 5% and 50% [5], [6], depending on the type of insomnia (ie, acute and chronic), population studied (eg, women or men; young or elderly), and assigned definition (ie, from symptoms of insomnia through to meeting diagnostic criteria).

Previous studies have identified risk factors for insomnia, with female gender and increased age identified as predisposing demographic risk factors for insomnia in the general population [7], [8], [9], [10]. Other risk factors include psychiatric disorders, particularly depression, anxiety and personality disorders [11], physical ill-health [12], [13], stressful events [14], certain types of prescription medication [15], and substance misuse [16].

Prisoners have a higher prevalence of most of these precipitating factors compared to the general population [17], [18], [19]. In addition, the prison regime and environment may further interfere with the sleep–wake cycle due to the interruption of usual daily routines [20], excessive time in cell and lack of personal autonomy [21]. Lack of control over the physical environment are also likely to cause further disturbance, including experiencing too much or too little light [22]; excessive noise [22], [23] and uncomfortable furnishings [24].

There are significant gaps in the literature pertaining to insomnia in prison. A recent systematic integrative review of both quantitative and qualitative studies into insomnia in prison reported on the findings from 33 papers [25]. Prevalence rates ranged from 11% to 81%; however studies were heterogeneous in terms of methodologies employed, sample size and jurisdiction. No studies examined insomnia disorder (ID) prevalence in a prison setting. The authors proposed prevalence rates of insomnia using validated measures were needed to ensure they are measuring what is indicated (ie, insomnia).

Insomnia (in agreement with the general population) was more common in women prisoners; however, this was based on only three studies reporting gender-specific prevalence rates [12], [26], [27]. Some studies used the Pittsburgh Sleep Quality Index (PSQI) [28], a recommended measure for sleep quality and insomnia symptoms [29], but no studies utilised a tool designed to formally diagnose insomnia based on Diagnostic and Statistical Manual of Mental Disorders, fifth edition (DSM-V) or International Classification of Sleep Disorders (ISCD-2) criteria such as the Insomnia Severity Index (ISI) [30] or Sleep Condition Indicator (SCI) [31]. The review concluded that definitive data on prevalence, gender differences and risk factors are still lacking.

In this paper we will establish the prevalence of insomnia in several English prisons and identify demographic, clinical and environmental factors to inform service delivery.

## 2 Methods

### 2.1 Participants and setting

Prisoners were randomly sampled from two prisons for adult men (one category B local prison which serve local courts, accepting sentenced and newly convicted prisoners or those serving short sentences and one category C training which houses men part way through longer sentences in the North West^1^) and one prison for young and adult women in North East England from January 2013 through April 2014. Inclusion criteria were prisoners over 18 and able to consent for themselves. The exclusion criteria were: being unable to provide informed consent due to being too physically or mentally unwell, presence of risk markers that indicated that the interview could not be conducted alone, and being a non-English speaker. No additional reward was given to prisoners from participating in the study. However, interviews were arranged to avoid overlap with their participating activities. The study was approved by the National Health Service (NHS) Research Ethics Committee for Wales (ref: 13/WA/0249); the National Offender Management Service (ref: 2013-208); local research governance boards; and prison governors from each participating prison. All participants gave informed written consent.

### 2.2 Sample size calculation

The sample size was calculated using the identification of insomnia as the main outcome. Ascertaining a precise prevalence benchmark of insomnia was difficult due to reported wide prevalence (11.8% and 81.0%) and variation in insomnia definitions and methodologies used across previous studies [25]. Therefore, for the purpose of estimating sample size, we assumed the prevalence rate of insomnia to be 50% (the value that would produce the highest possible sample size). On this basis, a total of 92 participants were needed to estimate the proportion of prisoners with insomnia among a prison population of 1826,^2^ with a 10% margin of error and a 95% confidence interval. To perform secondary analyses, increasing the sample to 202^3^ allowed the detection of differences in key predictors between those with and without insomnia. To further guard against a possibly higher than anticipated dropout rate, the final sample required was increased to 240.

### 2.3 Protocol

Data collection was carried out across the three sites concurrently; each prison could accommodate the research on a limited number of days each week, and therefore working across all three making the best use of the researcher's time. A census day was chosen for each site on which a list of all current prisoners was obtained and prisoner names and cell locations only were transferred to a Microsoft Excel spreadsheet. Using a random number generator formula, a list of prisoners to be approached for inclusion was created. When all prisoners on the list had been approached, another list was generated and the process repeated until the required number of participants was achieved.

Prison officers initially approached potential participants to determine interest in talking to the researcher about the study. For each prisoner who expressed interest in taking part, a provisional interview date and private interview room was organised for at least 24 h to give them time to decide whether they still wanted to take part. A questionnaire battery was then administered with each participant face-to-face to ensure clarity and understanding of questions.

### 2.4 Measures

Each interview comprised a battery of validated sleep measures and one tool specifically designed for this study.

### 2.5 Sleep measures

The Sleep Condition Indicator (SCI) [31] is an eight-item screening tool used for further evaluation of possible ‘insomnia disorder’ (ID) using a clinical interview. The term ID will be used henceforth. The SCI is unique in appraising symptoms specifically against DSM V diagnostic criteria for ID [2], including sleep continuity; sleep satisfaction, severity of symptoms, and daytime consequences of poor sleep. Items are rated on a 0–4 scale with a total score of ≤16 indicating possible ID. Excellent reliability (α = 89) and good concurrent validity have been demonstrated for the SCI [31], [32].

The Pittsburgh Sleep Quality Index (PSQI) [28] assesses subjective sleep quality. The 19 items encompass seven domains including sleep latency (the time taken to fall asleep), sleep duration, sleep quality, efficiency, sleep disturbances, use of sleeping medication and daytime dysfunction. Each item is scored zero to three and the sum allows for a subjective sleep quality score. A cut off score of more/greater than five distinguish ‘poor sleepers’ from ‘good sleepers’ (≤5). The PSQI has excellent reliability (α = 89) and has been recommended for measuring insomnia symptoms [29].

The Sleep Disorders Screener (SDS) [33] is a short tool that identifies sleep disorders other than insomnia, used in clinical practice. It screens for narcolepsy, circadian rhythm sleep disorder, parasomnia, sleep breathing disorder, and periodic limb movements of sleep/restless legs syndrome (PLMS/RLS). Internal consistency is excellent (α = 0.79).

The Sleep Hygiene Index (SHI) [34] is a 13 item tool to measure sleep hygiene practice. Each item reflects an element of poor sleep hygiene, for example coffee before/after going to bed, irregular sleep onset and awakening,; and daytime nap. One item was changed by removing references to alcohol, to reflect the prison environment. Test-retest reliability (*r* (139) = 0.71, p < 0.01) and validity is good [34].

The Dysfunctional Beliefs and Attitudes about Sleep (DBAS-16) [35] scale comprises 16 statements rated on a Likert scale of 0–10, with zero being strongly disagree and 10 strongly agree. An average score is obtained from all the questions. Each statement reflects beliefs and attitudes about sleep, with higher scores indicative of dysfunctional beliefs. Reliability of the measure is also excellent (α = 0.79).

### 2.6 Demographic, forensic and clinical measures

The Brief Psychiatric Rating Scale (BRPS) [36] is a commonly used measure of a wide range of individual psychiatric symptoms, rather than being a diagnostic tool for one or more specific diagnoses. There are 24 items that represent symptom constructs including hostility, anxiety and psychotic symptoms. Each is scored on a scale of 1 (not present) to 10 (extremely severe), relating to the previous two weeks. Thresholds of clinical severity are given for each item, to be explored through semi-structured interview. Items scores can be divided into absent, present at clinical level and present but sub-clinical; reliability is excellent [36].

Because no measure to assess the impact of the prison environment on sleep existed, one, named the Prison Environment Sleep Questionnaire (PESQ), was designed especially for this study. The PESQ is a 16-item measure to capture elements of the prison environment likely to disturb sleep. Items were developed, defined and selected on the basis of previous literature [37], [38] and through consultations with prison based clinicians and an ex-prisoner. Factors such as noise, temperature, light or psychological issues (e.g., being worried or anxious, thinking too much) were included. Reliability tests showed it had a strong internal consistency (α = 0.83).

A pro-forma questionnaire to capture basic demographic information, including age, gender and ethnicity, lifetime substance misuse, and any physical health conditions known to be associated with insomnia (e.g., chronic pain, gastrointestinal problems etc.) was also created and administered.

### 2.7 Statistical analysis

Descriptive data were used to describe demographic, forensic and clinical characteristics. The prevalence of insomnia was estimated using counts, proportions and 95% confidence intervals (CI). Chi-square and t-tests were used to determine associations between dichotomous and continuous data, respectively. Multivariable logistical regression techniques adjusted for gender and age were performed to identify predictors of insomnia, with demographic, clinical, and prison-related factors entered as independent variables. To ensure the best model fit, we firstly included all predictors previously associated with insomnia in the literature including physical ill-health, sleep hygiene and all psychiatric symptoms (eg, depression, suicidality etc.) measured using individual BPRS items. We then added prison-related factors to the model, such as prisoner status and prison environmental factors. We reviewed and reduced the model systematically at each stage based on the authors' judgement and variance explained (R^2^), eliminating individual predictors where they did not contribute to the full model, irrespective of significance in univariable analyses. Data management and analysis were performed using SPSSv22 [39].

## 3 Results

### 3.1 Demographic, forensic and clinical characteristics

**Fig. 1:**
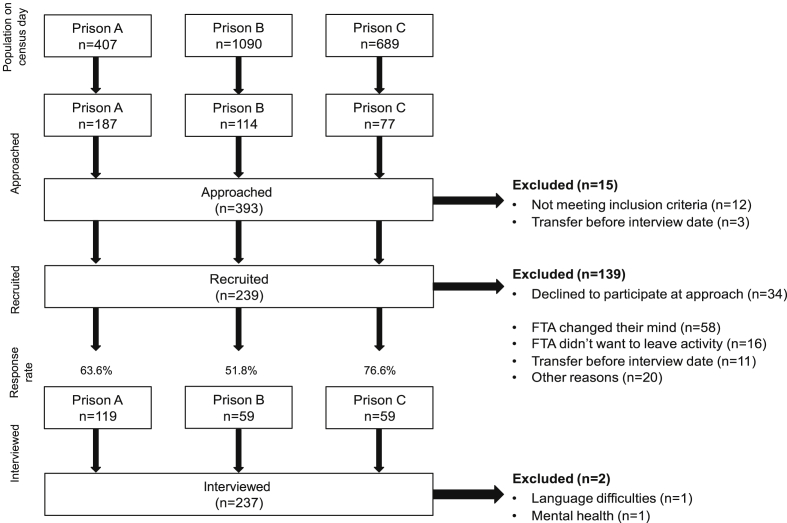
Flowchart of recruitment. FTA, failed to attend.

Two hundred and thirty-nine prisoners gave written consent. Fig. 1 shows reasons for non-attendance. The required sample size was achieved as 118 men and 119 women completed full interviews. Table 1 shows the demographic and forensic characteristics. Mean age was 36.2 (±11.9) and less than a fifth were from Black, Asian and minority ethnic backgrounds (BAME) (13.9%; n = 33). The average duration of time in prison was 25.4 months (±37.9), ranging from less than a month to 30 years.

### 3.2 Prevalence of insomnia and sleep quality

**Fig. 2:**
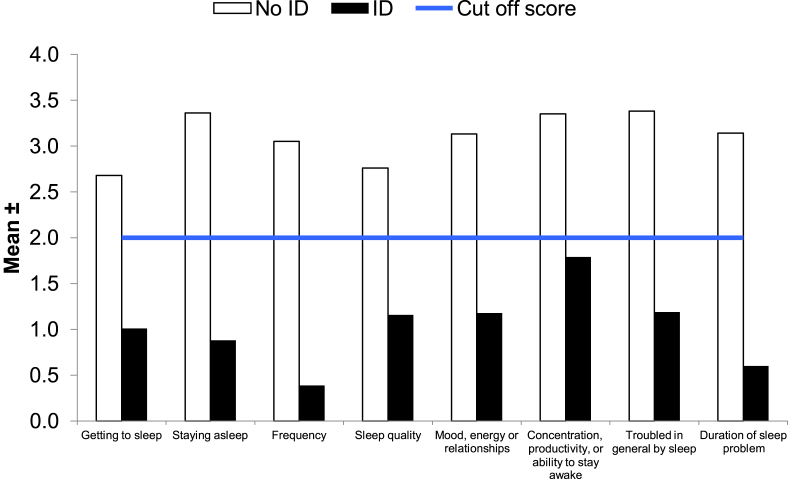
SCI components among prisoners with and without ID. SCI, sleep condition indicator; ID, insomnia disorder. *p < 0.001.

Using stringent DSM-V criteria (as measured by the SCI), 61.6% (95% CI, 55.4%–67.8%) prisoners had ID. Nearly two-thirds of prisoners with ID took longer than 60 min to initiate sleep (61.0%; CI, 53.1%–68.9%). Most prisoners with ID were classified as chronic (≥3 months; 89.7%; CI, 84.8%–94.6%) and had it for more than three nights a week (95.2%; CI, 91.7%–98.7%). Individually, SCI component scores for prisoners with ID were all significantly lower than prisoners without ID, which indicated the presence of distinct symptoms of insomnia (Fig. 2).

**Fig. 3:**
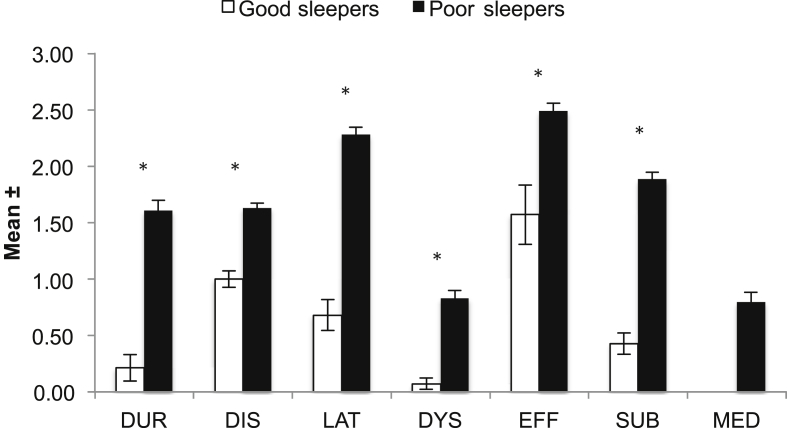
PSQI components by sleep type. DUR, duration; DIS, sleep disturbance; LAT, sleep latency; DYS, daytime dysfunction; EFF, sleep efficiency; SUB, subjective sleep quality; MED, sleep medication. *p < 0.001.

The prevalence of those who subjectively regarded themselves as “poor sleepers”, as measured by the PSQI, was 88.2% (CI, 84.1%–92.3%). Scores for all PSQI items were significantly higher among poor sleepers than good sleepers. In particular, sleep latency mean scores were significantly higher for poor sleepers than good sleepers (2.28 vs. 0.68; t (235) = −8.570, p < 0.001) (Fig. 3).

### 3.3 Gender differences in insomnia and sleep quality

ID was significantly more prevalent in women (70.6%; CI, 62.4%–78.8%) than men (52.5%; CI, 43.5%–61.5%) (χ2 = 8.157; p < 0.005). Women also reported significantly more problems initiating sleep (1.38 vs 1.92; t (232) = 2.543, p < 0.05) and their mood, energy or relationships were more negatively affected by poor sleep than men (1.61 vs 2.26; t (235) = 3.419, p < 0.01).

### 3.4 Clinical factors associated with insomnia

Clinical characteristics and sleep-related symptoms of prisoners with and without ID are summarised in Table 2. No significant differences were found between ID and no ID groups on lifetime substance misuse, except lifetime amphetamine and heroin use (Table 2). Prisoners with ID were significantly more likely than the no ID group to have previously sought help for insomnia, had pain in the last month, and reported a history of physical ill-health (Table 2). Prisoners with ID had significantly higher mean scores on poor sleep hygiene and were more likely to endorse dysfunctional beliefs about sleep. Moreover, prisoners with ID had significantly higher BPRS scores, reflecting more severe subclinical mental health problems (Table 2). Notably, prisoners with ID were significantly more likely to exhibit symptoms of anxiety, depression, suicidality and suspiciousness.

### 3.5 Prison-related environmental factors of insomnia

The PESQ also revealed that overall mean scores of prison-related situational factors were significantly higher in those with ID (p < 0.001) (Table 3). Similarly, mean scores for several individual items were significantly higher for those with ID, including uncomfortable mattresses, being too hot and noise.

## 4 Multivariable factors of insomnia

A multivariable logistic regression analysis was performed to identify risk factors for insomnia in prison (Table 4). After adjusting for gender and age, non-retained variables included suspiciousness, disorientation, physical pain, prisoner status, lifetime heroin use, index offence, prisoner status, previous number of times in prison and duration of time in prison. Nine variables remained: history of physical ill-health, previous help-seeking for insomnia, depression, anxiety, suicidality, dysfunctional beliefs and attitudes about sleep, poor sleep hygiene, other sleep disorders and problematic prison environment. Of particular significance were history of physical ill-health, suicidality, previous help for insomnia and depression. The full model was significant (*χ*^2^ (6, n = 237) = 107.703, p < 0.001).

## 5 Discussion

### 5.1 Main findings and comparison to other studies

This study is the first to estimate prevalence and predictors of ID in adult prisoners in England. Perhaps the most striking finding is that, overall, two-thirds of the sample had possible ID (61.6%) compared with 45.7% of a large UK general population sample using the same measure (SCI) [32]. In our study, 70.6% of female prisoners had possible ID, which is comparable to a previous prison study, despite their less stringent use of DSM-V criteria [12]. In line with previous studies conducted in the wider community, we found that physical ill-health, dysfunctional beliefs about sleep, poor sleep hygiene and depression were all significantly associated with insomnia [40], [41]. Additionally, results infer prison environmental factors and suicidality are also associated with insomnia in prison.

Insomnia is a symptom of depression worldwide [42]. There is very strong evidence of an association between depression and suicide; indeed, risk of suicide completion has a 20-fold increase when the individual has experienced a major depressive episode [43]. This relationship posits increased likelihood that the relationship between suicide and insomnia may be due to depression. This may be because of the complexity between suicide, depression and insomnia [44]. However, there is increasing evidence of an association between insomnia and suicidality in the general and prison populations [45], [46], [47], [48]. Furthermore, in a sample of 1420 prisoners, insomnia significantly predicted suicidality, independent of depression [47]. Our findings also highlighted an association between insomnia and suicidality and insomnia and depression separately in a prison population. However, we are only the second study to examine the relationship between suicide and insomnia in prison therefore, more research is needed to verify the association.

Community studies have found substance misuse to be associated with insomnia [16], [49]; however, in our study there was no relationship between all-encompassing illicit substance misuse and insomnia in prison. Individual drugs, heroin and amphetamine use were significantly associated with insomnia in our univariable analyses, but they were not retained as a predictor in multivariable analyses. This contrasts with previous studies, which have more strongly identified substance misuse as a predictor of insomnia. This discrepancy could be due to methodological differences, such as limited specificity in our measure. Alternatively, it could indicate that the relationship is more complex than first thought. Most prison studies into insomnia to date have focused on one or two factors; only two studies looked at a range of factors in a holistic manner [50], [51]. Both studies found substance misuse predicted insomnia, in contrast to our study. However, these studies relied on data gathered retrospectively from clinical notes describing physician consultations, a method, which arguably lacks consistency and construct validity, potentially underestimating insomnia.

### 5.2 Assessing the prevalence of insomnia

The findings from our integrative review proposed prevalence rates of ID using validated measures. The SCI is a robust diagnostic tool utilising DSM-V criteria for ID and the PSQI measures insomnia symptoms, both validated on several populations. In this study, overall prevalence of ID according to the SCI [31] was 61.6%; and 88.2% of the sample experienced subjectively poor sleep using the PSQI [28]. Estimates of the former tool are valid, but the latter has previously been used successfully in a prison environment [51] and community, which allows for comparison of symptoms across studies. Based on this combination, we can be confident that insomnia is common in prison, however the gold standard full independent clinical interview would be needed to further verify these results.

### 5.3 Implications

The high prevalence of insomnia suggests it is a public health concern in prisons, which highlights the need for treatment attention, particularly because of its association with other pertinent conditions in prison such as mental and physical ill health. Prisoners are generally more likely to have chronic physical conditions, four times more likely to have a mental health condition and seven times more likely to commit suicide than the general population [18], [52], [53]. Notably, suicide is four times more likely to occur at night, a time when prisoners are locked down, and staff are limited, therefore regular checks are reduced [54]. Being awake at night may therefore represent vulnerability for completed suicide in prison. Knowing that insomnia is linked to physical and mental health conditions could explain why these conditions are common in prison which points towards the need for appropriate screening, assessment and treatment of insomnia in prison. Identifying predisposing, precipitating and perpetuating factors underlying insomnia in prison can help target treatments [55] and may offer potential for early detection and prevention of insomnia. In particular, women are more likely to have insomnia (predisposing) and prisoners with insomnia may exhibit symptoms of depression and suicidality (precipitating) and have maladaptive beliefs about sleep and poor sleep hygiene (perpetuating), which can help to inform service delivery. These study findings alongside wider evidence could be used as the basis of a clinical pathway to help effectively manage insomnia in prison.

### 5.4 Limitations

Several limitations to this study need to be acknowledged. First, as a cross-sectional design was utilised, no conclusions can be made about causality. The history of previous help for insomnia may suggest the presence of lifetime history of insomnia (pre-existing insomnia may predispose prisoners to insomnia before prison admission). Second, results were based on survey responses and therefore may potentially be subject to self-report bias. Nevertheless, the subjective nature of the assessment is applicable and likely in congruence with day-to-day healthcare consultations (so it is ecologically comparable). Despite this, objective measures such as PSG (ie, physiological recording of awake and rest activity) or actigraphy (ie, a wrist like device that measures awake and rest activity) could further elucidate findings. Third, while the overall sample size was reasonable, lack of power in some sub-analyses may have explained some borderline significant results, incurring a potential risk of type-II statistical error. Fourthly, the PESQ is not validated, therefore results should be examined with caution. Generalizability may be limited due to sampling issues. For instance, on approach some prisoners were excluded, such as prisoners without capacity to consent due to severe mental and physical health problems. We emphasized that participation was not dependent on being a poor sleeper; however, it is possible that those with no sleep problems excluded themselves. This may have resulted in underrepresentation of good sleepers. However, only 9% (n = 34) of prisoners declined to participate on approach. Moreover, only one female prison, category B local and category C training prisons participated, representing around 70% of the England and Wales' prison population. We did not sample prisoners from category A, high secure, category D, open prisons or anyone under 18 years old. Our results therefore may not be generalizable to the entire England and Wales prison estate and need to be interpreted with caution.

## 6 Conclusion

This study has identified the prevalence of insomnia and examined associated factors in prison populations. Notwithstanding limitations, the study suggests that the prevalence for possible ID is higher in prisons than in the general population. A range of associated factors for insomnia were identified, most notably a previous history of physical-ill health, and/or receiving help for insomnia, suicidality and depression. Poor sleep hygiene, maladaptive beliefs about sleep and the problematic prison environment may maintain symptoms of insomnia in prisoners. The current study findings are relevant to informing clinical practice in the screening, assessment and treatment of insomnia. Indeed, our study, alongside wider evidence, could be used as the basis of a clinical pathway to help effectively manage insomnia in prison. Future research is needed to further validate these findings on a larger-scale, using objective measures for sleep.

## Institution

Work was performed in The University of Manchester.

## Disclosure of presence of financial support

Lindsay Dewa is supported by Medical Research Council award 1233315.

## Figures and Tables

**Table 1:** Demographic and forensic characteristics of prisoners (n = 237).

	Prison A	Prison B	Prison C	Total (N, %)
**Gender**				
Men	0	59	59	118 [49.7]
Women	119	0	0	119 [50.2]
**Age group**				
18–24	21 [17.6]	9 [15.3]	10 [16.9]	40 [16.9]
24–29	16 [13.4]	15 [25.4]	11 [18.6]	42 [17.7]
30–44	58 [48.7]	17 [28.8]	26 [44.1]	101 [42.6]
45–64	21 [17.6]	14 [23.7]	10 [16.9]	45 [19.0]
65+	3 [2.5]	4 [6.8]	2 [3.4]	9 [3.8]
**Ethnicity**				
White-British	100 [84.0]	48 [81.4]	56 [94.9]	204 [86.1]
Asian or Asian	4 [3.4]	3 [5.1]	1 [1.7]	8 [3.4]
British Black or Black British	5 [4.2]	5 [8.5]	1 [1.7]	11 [4.6]
Mixed Background	5 [4.2]	1 [1.7]	1 [1.7]	8 [3.4]
Other	5 [4.2]	2 [3.4]	0	6 [2.5]
**Marital status**				
Single	88 [73.9]	42 [71.2]	47 [79.7]	177 [74.7]
Married	18 [15.1]	4 [6.8]	4 [6.8]	26 [11.0]
Divorced	4 [3.4]	12 [20.3]	6 [10.2]	22 [9.3]
Other	9 [7.6]	1 [1.7]	2 [3.4]	12 [5.1]
**Index offence**				
Violent offence	32 [26.9]	22 [37.3]	12 [20.3]	66 [27.8]
Robbery offence	15 [12.6]	6 [10.2]	12 [20.3]	33 [13.9]
Burglary	10 [8.4]	10.2 [10.2]	10 [16.9]	26 [11.0]
Sexual offence	9 [7.6]	14 [23.7]	4 [6.8]	27 [11.4]
Drug offence	6 [5.0]	5 [8.5]	11 [18.6]	22 [9.3]
**Prisoner status**				
Sentenced	110 [94.4]	59 [100.0]	43 [72.9]	212 [89.5]
Remand	8 [6.7]	0 [0.0]	13 [22.0]	21 [8.9]
Un-sentenced	1 [0.8]	0 [0.0]	3 [5.1]	4 [1.7]
Previous number of times in prison (mean, SD)	3.0 [7.8]	2.7 [5.5]	5.3 [8.2]	3.5 [7.4]
Duration of time in prison (months) (mean, SD)	22.1 [29.3]	47.0 [56.7]	9.7 [10.4]	25.4 [37.9]

**Table 2:** Clinical and sleep-related characteristics for prisoners by ID status.

	ID (n = 146)	No ID (n = 91)	Statistic	P value
**Clinical characteristics**				
*Prior service lifetime use*				
Previous help for insomnia (N, %)	104 [71.2]	31 [34.1]	31.588	<0.001
*History of physical ill health*				
Reported pain in last month (N, %)	96 [65.8]	31 [34.1]	22.632	<0.001
Reported history of physical ill-health (N, %)	124 [84.9]	64 [70.3]	7.288	0.007
*Reported drug use (lifetime)*				
Amphetamines (N, %)	75 [51.4]	35 [38.5]	3.756	0.053
Heroin (N, %)	58 [39.7]	25 [27.5]	3.699	0.054
*Recent psychiatric symptoms (BPRS)*				
Anxiety (mean, SD)	2.7 [1.4]	1.7 [0.9]	−6.684	<0.001
Depression (mean, SD)	2.7 [1.5]	1.5 [0.8]	−7.845	<0.001
Disorientated (mean, SD)	1.1 [0.5]	1.0 [0.2]	−2.330	0.034
Elevated mood (mean, SD)	1.4 [1.0]	1.2 [0.6]	−2.596	0.021
Guilt (mean, SD)	1.9 [1.2]	1.4 [0.8]	−4.100	<0.001
Hostility (mean, SD)	1.9 [1.2]	1.5 [0.8]	−4.422	0.001
Somatic concern (mean, SD)	1.8 [1.2]	1.3 [0.6]	−3.531	<0.001
Suicidality (mean, SD)	1.4 [1.0]	1.0 [0.2]	−4.120	<0.001
Suspiciousness (mean, SD)	2.0 [1.0]	1.3 [0.7]	−5.740	<0.001
Tension (mean, SD)	1.5 [0.7]	1.3 [0.6]	−2.052	0.041
Unusual thought content (mean, SD)	1.4 [0.9]	1.1 [0.6]	−2.729	0.007
BPRS (mean, SD)	35.4 [7.5]	30.1 [5.3]	−6.442	<0.001
**Sleep-related beliefs and symptoms**				
SDS (N, %)	66 [45.2]	16 [17.6]	18.903	<0.001
SHI (mean, SD)	32.7 [7.5]	27.0 [6.2]	−6.140	<0.001
DBAS-16 (mean, SD)	5.7 [1.9]	3.8 [1.8]	−7.546	<0.001
PSQI (mean, SD)	12.9 [3.2]	6.9 [2.6]	26.887	<0.001
SCI (mean, SD)	8.2 [4.5]	24.9 [4.8]	−15.315	<0.001

BPRS, Brief Psychiatric Rating Scale; SHI, Sleep Hygiene Index; DBAS-16, Dysfunctional Beliefs and Attitudes about Sleep Scale; PESQ, Prison Environmental Sleep Questionnaire; PSQI, Pittsburgh Sleep Quality Index; SCI, Sleep Condition Indicator; SD, standard deviation; SDS, other sleep disorder.

**Table 3:** PESQ components for prisoners by ID status.

PESQ component	ID (n = 146)	No ID (n = 91)	Statistic	P value
Worried or anxious (mean, SD)	2.5 [1.5]	1.0 [1.2]	−8.427	<0.001
Mind was racing (mean, SD)	2.5 [1.5]	1.0 [1.3]	−8.734	<0.001
Noise from doors slamming (mean, SD)	1.8 [1.5]	1.1 [1.4]	−3.547	<0.001
Noise from the prison environment (mean, SD)	1.6 [1.7]	0.9 [1.2]	−3.943	<0.001
Prisoner sounds (mean, SD)	1.3 [1.5]	0.7 [1.1]	−3.825	<0.001
Noise from staff (mean, SD)	1.3 [1.5]	0.8 [1.3]	−2.529	0.012
Water noise (mean, SD)	1.1 [1.5]	0.6 [1.2]	−0.2580	0.011
Noise from the TV and/or radio (mean, SD)	1.0 [1.1]	0.6 [1.4]	−0.247	0.026
Noise from intercom or telephone (mean, SD)	0.8 [1.3]	0.6 [1.1]	−1.357	0.176
Bed parts squeaking (mean, SD)	0.7 [1.2]	0.4 [0.9]	−1.927	0.055
Mattress was too uncomfortable (mean, SD)	2.4 [1.7]	1.5 [1.8]	−4.131	<0.001
Physical pain (mean, SD)	1.6 [1.6]	0.5 [1.0]	−6.635	<0.001
Too hot (mean, SD)	1.5 [1.6]	0.8 [1.2]	−3.891	<0.001
Too cold (mean, SD)	1.1 [1.5]	0.6 [1.0]	−2.519	0.012
Too light in my cell (mean, SD)	0.8 [1.3]	0.3 [0.9]	−3.291	<0.001
Prisoner incidents^a^ (mean, SD)	0.4 [0.8]	0.3 [0.7]	−1.485	0.139
PESQ total (mean, SD)	22.3 [11.5]	11.6 [9.1]	−7.947	<0.001

a Including a violent incident, general prisoner disturbance etc.

**Table 4:** Summary of multivariable logistic regression with clinical and forensic factors of insomnia.

Factor	Logistic coefficient	Odds ratio (OR)	95% CI	P value
Reported history of physical ill-health	1.286	3.62	1.31–9.98	0.013
BPRS Suicidality	1.024	2.79	1.01–7.66	0.047
Previous help for insomnia	0.946	2.58	1.21–5.47	0.014
BPRS Depression	0.723	2.06	1.31–3.24	0.002
DBAS-16	0.405	1.50	1.21–1.87	<0.001
SDS	0.308	1.36	0.57–3.25	0.488
SHI	0.103	1.11	1.04–1.19	0.003
PESQ	0.069	1.07	1.02–1.12	0.003
BPRS Anxiety	−0.355	0.70	0.43–1.14	0.153
Nagelkerke R^2^ .59				

BPRS, Brief Psychiatric Rating Scale; SHI, Sleep Hygiene Index; DBAS-16, Dysfunctional Beliefs and Attitudes about Sleep Scale; PESQ, Prison Environmental Sleep Questionnaire; SDS, other sleep disorder; CI, confidence interval.

## Footnotes

1 In England and Wales prisons for adult men are categorised in terms of their security with category A prisons being the most secure through to category D which are open prisons. Most recent population figures show that 38.3% of adult men are held in cat B and 25.5% in category C conditions. Just under 5% of the prison population are women. We sampled approximately 9.3% of the overall women's prison population.

2 Total population on census days across three prisons.

3 Identified as the number needed from sample size calculation.
